# SNP Variants in Major Histocompatibility Complex Are Associated with Sarcoidosis Susceptibility—A Joint Analysis in Four European Populations

**DOI:** 10.3389/fimmu.2017.00422

**Published:** 2017-04-19

**Authors:** Annika Wolin, Elisa Laura Lahtela, Verneri Anttila, Martin Petrek, Johan Grunewald, Coline H. M. van Moorsel, Anders Eklund, Jan C. Grutters, Vitezslav Kolek, Frantisek Mrazek, Amit Kishore, Leonid Padyukov, Anne Pietinalho, Marcus Ronninger, Mikko Seppänen, Olof Selroos, Marja-Liisa Lokki

**Affiliations:** ^1^Transplantation Laboratory, Medicum, University of Helsinki, Helsinki, Finland; ^2^Analytical and Translational Genetics Unit, Department of Medicine, Massachusetts General Hospital, Harvard Medical School, Boston, MA, USA; ^3^Program in Medical and Population Genetics, Broad Institute of MIT and Harvard, Cambridge, MA, USA; ^4^Institute for Molecular Medicine Finland (FIMM), University of Helsinki, Helsinki, Finland; ^5^Department of Pathological Physiology and Institute of Molecular and Translational Medicine, Faculty of Medicine and Dentistry, Palacky University Olomouc, Olomouc, Czech Republic; ^6^Respiratory Medicine Unit, Department of Medicine Solna and CMM, Karolinska Institutet, Karolinska University Hospital, Solna, Sweden; ^7^Department of Pulmonology, St. Antonius Hospital Nieuwegein, Heart and Lung Center, University Medical Center Utrecht, Utrecht, Netherlands; ^8^Department of Respiratory Medicine, Faculty of Medicine and Dentistry, Palacky University Olomouc, Olomouc, Czech Republic; ^9^Department of Immunology, Faculty of Medicine and Dentistry, Palacky University Olomouc, Olomouc, Czech Republic; ^10^Department of Pathological Physiology, Faculty of Medicine and Dentistry, Palacky University Olomouc, Olomouc, Czech Republic; ^11^Rheumatology Unit, Department of Medicine, Karolinska Institutet, Karolinska University Hospital, Stockholm, Sweden; ^12^Raasepori Health Care Centre, Raasepori, Finland; ^13^Rare Disease Center, Children’s Hospital and Adult Immunodeficiency Unit, Inflammation Center, Helsinki University and Helsinki University Hospital, Helsinki, Finland; ^14^University of Helsinki, Helsinki, Finland

**Keywords:** *BTNL2*, SNP, *DRB1*, HLA, prognosis, sarcoidosis, major histocompatibility complex, haplotype

## Abstract

Sarcoidosis is a multiorgan inflammatory disorder with heritability estimates up to 66%. Previous studies have shown the major histocompatibility complex (MHC) region to be associated with sarcoidosis, suggesting a functional role for antigen-presenting molecules and immune mediators in the disease pathogenesis. To detect variants predisposing to sarcoidosis and to identify genetic differences between patient subgroups, we studied four genes in the MHC Class III region (*LTA, TNF, AGER, BTNL2*) and *HLA-DRA* with tag-SNPs and their relation to *HLA-DRB1* alleles. We present results from a joint analysis of four study populations (Finnish, Swedish, Dutch, and Czech). Patients with sarcoidosis (*n* = 805) were further subdivided based on the disease activity and the presence of Löfgren’s syndrome. In a joint analysis, seven SNPs were associated with non-Löfgren sarcoidosis (NL; the strongest association with rs3177928, *P* = 1.79E−07, OR = 1.9) and eight with Löfgren’s syndrome [Löfgren syndrome (LS); the strongest association with rs3129843, *P* = 3.44E−12, OR = 3.4] when compared with healthy controls (*n* = 870). Five SNPs were associated with sarcoidosis disease course (the strongest association with rs3177928, *P* = 0.003, OR = 1.9). The high linkage disequilibrium (LD) between SNPs and an *HLA-DRB1* challenged the result interpretation. When the SNPs and *HLA-DRB1* alleles were analyzed together, independent association was observed for four SNPs in the *HLA-DRA/BTNL2* region: rs3135365 (NL; *P* = 0.015), rs3177928 (NL; *P* < 0.001), rs6937545 (LS; *P* = 0.012), and rs5007259 (disease activity; *P* = 0.002). These SNPs act as expression quantitative trait loci (eQTL) for *HLA-DRB1* and/or *HLA-DRB5*. In conclusion, we found novel SNPs in *BTNL2* and *HLA-DRA* regions associating with sarcoidosis. Our finding further establishes that polymorphisms in the *HLA-DRA* and *BTNL2* have a role in sarcoidosis susceptibility. This multi-population study demonstrates that at least a part of these associations are *HLA-DRB1* independent (e.g., not due to LD) and shared across ancestral origins. The variants that were independent of *HLA-DRB1* associations acted as eQTL for *HLA-DRB1* and/or *-DRB5*, suggesting a role in regulating gene expression.

## Introduction

Sarcoidosis (MIM 609464) is a multiorgan inflammatory disorder of unknown etiology. The majority of the sarcoidosis patients (of European descent) have a favorable prognosis if involvement is solely pulmonary, but the clinical picture and prognosis vary (1). Current understanding views sarcoidosis primarily as a multifactorial disorder with heritability estimates up to 66%, with possible environmental triggers in addition to the susceptibility gene(s) (2–4).

Sarcoidosis is manifested by accumulation of activated CD4-positive T lymphocytes and macrophages at disease sites suggesting a functional role for antigen-presenting molecules and immune mediator genes (4). The search for genetic components, indicated on the basis of family and multi-ancestral studies (2, 5), has shown a strong role of the major histocompatibility complex (MHC) region at chromosome 6p21.3 in susceptibility to sarcoidosis. Recently, several genome-wide association studies have indicated other regions of interest as well (6–8), but none as influential as the MHC.

Clinical manifestations in sarcoidosis range from asymptomatic disease to severe loss-of-function, including an acute disease (Löfgren’s syndrome), which usually resolves spontaneously, a subacute disease, which also may resolve spontaneously or with treatment, and a chronic/progressive disease. Associations vary from protective to predisposing markers, or markers influencing clinical outcomes. Thus, previous studies have pointed out the importance to characterize patient groups according to clinical phenotypes to avoid inconsistency between studies (6, 9–12). Associations with sarcoidosis also vary between different ancestral groups. Most classical examples being strong association found between HLA-DRB1*03 and Löfgren’s syndrome in caucasian populations, whereas among the Japanese, this association is absent due the lack of HLA-DRB1*03 allele in the population (11).

The most probable pathophysiology of sarcoidosis, the dysregulation of the immune response (13), strongly suggests benefits from a better understanding of the role of the immune-mediating genes in sarcoidosis susceptibility. Based on the robust evidence of the MHC class III gene association to sarcoidosis, this particular region warrants further investigation. Probably, the most investigated variant in the MHC class III, the splice-site SNP in *BTNL2* (OMIM 606000) gene (rs2076530 G>A), has so far shown contradictory results with different sarcoidosis phenotypes and populations (7, 14–16).

Here, we present results from a Finnish case-control discovery sample as well as three independent replication studies from the Swedish, Dutch, and Czech populations. Our aim was to investigate genetic variance and phenotype specific variants in five functional candidate genes: lymphotoxin alfa (*LTA*; *OMIM 153440)*, tumor necrosis factor (*TNF*; *OMIM 191160)*, advanced glycosylation end product-specific receptor (*AGER*; *OMIM 600214)*, butyrophilin-like 2 (*BTNL2*; *OMIM 606000)*, and HLA-DR alpha (*HLA-DRA*; *OMIM 142860)* within the MHC classes III and II with tag-SNP genotyping approach. We also address the question whether the tag-SNP associations were due to the strong linkage disequilibrium (LD) with *HLA-DRB1*. Replication of gene variants predisposing to disease and disease phenotypes in several European populations would indicate their important role in disease development.

## Patients and Methods

### Patients and Control Subjects

Total of 805 sarcoidosis patients and 870 controls were included in the study. The discovery sample set consisted of 188 Finnish sarcoidosis patients and 150 Finnish healthy controls. Swedish (cases = 219, controls = 360), Dutch (cases = 180, controls = 180), and Czech (cases = 218, controls = 180) data sets were used for the replication and the joint analysis. In quality control, 40 sarcoidosis patients (1 Finnish, 29 Swedish, 10 Czech) and 11 controls (2 Swedish, 2 Czech, 7 Dutch) were excluded due to low quality of genotyping results.

Table 1 shows the sample characteristics. Although we had no age matched controls to the patients, the controls were unrelated healthy individuals recruited from the same geographic and ancestral background as the cases. More detailed sample characteristics and DNA extraction have been previously reported (10, 12, 17–19). Briefly, in the Finnish discovery sample, the patients were recruited from 17 pulmonary units throughout the country. The control population consisted of voluntary Finnish subjects attending a health survey, representing the Finnish population. The Swedish patients were recruited from single center, Karolinska University Hospital, Solna, Sweden. The control group consisted of consecutively collected Scandinavian blood donors. The Czech cases were gathered from the Olomouc University Hospital, Olomouc, Czech Republic, and controls were Czech nationality blood donors from the same region. The Dutch patients with sarcoidosis were from the St. Antonius Hospital, Nieuwegein, Netherlands. The control subjects comprised of healthy, Dutch Caucasian employees of the St. Antonius Hospital, and blood donors from Sanquin blood bank in the Netherlands. In all four study populations, the DNA was extracted from the buffy coat fraction of whole blood samples.

**Table 1:** **Sample characteristics**.

		NL sarcoidosis subgrouping after 2 years						
		Total (*n*)	Total (*n*) in genetic analysis	Individuals with NL (%)	Individuals with resolved sarcoidosis (NLR) (%)	Individuals with persistent sarcoidosis (NLP) (%)	Individuals with no subgroup info available (%)	Individuals with LS (%)
**Discovery sample**								
Finland	Sarcoidosis patients	188	187	89.8	42.2	47.6	0.0	10.2
	Controls	150	150					
**Replication sample**								
Sweden	Sarcoidosis patients	219	190	58.9	17.4	39.5	2.1	41.1
	Controls	360	358					
Dutch	Sarcoidosis patients	180	180	100.0	50.0	50.0	0.0	0.0
	Controls	180	173					
Czech	Sarcoidosis patients	218	208	81.3	22.6	39.9	18.8	18.8
	Controls	180	178					
**Combined discovery and replication sample**								
Total samples	Sarcoidosis patients	805	765	82.2	32.5	44.1	5.6	17.8
	Controls	870	859					

*The non-Löfgren sarcoidosis patients (NL) were subphenotyped into those with the disease resolved within 2 years (NLR) and those with persisting activity at that time point (NLP). Patients with Löfgren syndrome (LS) were not subgrouped*.

*n, number of subjects used in the SNP analysis (initial number of subjects)*.

Disease activity was determined using the generally accepted WASOG (World Association of Sarcoidosis and Other Granulomatous diseases) criteria. The sarcoidosis patients had been followed for at least 2 years and further subdivided into those with a resolved (normalized radiography and pulmonary lung function; no signs of active extrapulmonary disease) or a non-resolved disease (persistence of chest radiographic changes with clinical signs of disease activity). The resolution threshold in the Finnish, Swedish, and Czech populations was 2 years but 4 years in the Dutch. The patients were grouped into those with Löfgren’s syndrome [Löfgren syndrome (LS); *n* = 136] and non-Löfgren’s syndrome patients (NL; *n* = 629). Dutch cohort did not contain LS patients.

NL patients were grouped into those with a non-active disease [NL resolved (NLR); *n* = 249] and those with persisting activity at that time point [NL persistent (NLP); *n* = 337]. Forty-three patients (5.6%) had no subgroup information available, and we excluded them from the subgroup analysis. Due to small sample size, the LS patients were not subgrouped based on the disease activity.

All the subjects were of European descent and gave their written informed consent to participate in the genetic association study. The local Ethics Committee of the Department of Internal Medicine, Hospital District of Helsinki and Uusimaa, Finland approved the study protocol.

### Genotyping

We selected MHC gene regions of *HLA-DRA, LTA, TNF, AGER*, and *BTNL2* for SNP analysis and used the SNP tagging approach to identify a SNP or a set of SNPs associating with the trait. The tag-SNPs reduce the number of SNPs needed to cover the selected region and are chosen from the HapMap database. Information about the validation status, tagging quality, minor allele frequency (MAF) (>0.01), and gene structure was used for selecting the SNPs. In addition to HapMap database, the public dbSNP^1^ database was used to select additional SNPs. Eventually, 89 SNPs tagging two 100 and 92 SNP variants were genotyped in the Finnish discovery sample [*r*^2^ > 0.8; HapMap3 (20)]. The detailed information for SNP genotypes is provided in Table S1 in Supplementary Material. In the replication stage, 16 of the 89 SNPs (Table 2) were genotyped in the Swedish, Dutch, and Czech samples (Table S2 in Supplementary Material). Thirteen of the 16 SNPs were selected based on unadjusted *P*-values of *P* < 0.05 found in the discovery sample and only one SNP from each LD block (*r*^2^ < 0.8) were chosen. In addition, three SNPs (rs1800624, rs3130349, rs3135365) were included based on the published studies (14) showing sarcoidosis association.

**Table 2:** **Disease associated SNPs from the major histocompatibility complex region and their allele frequencies in the Finnish sarcoidosis discovery sample set**.

					Major allele frequency					Chi-square					
Marker	Candidate genes	Predicted function	Major allele	Minor allele	NL, *n* = 169	LS, *n* = 19	NLR, *n* = 79	NLP, *n* = 89	Controls, *n* = 150	NL vs. C, *P*-value	OR	LS vs. C, *P*-value	OR	NLR vs. NLP, *P*-value	OR
rs1800684	*AGER*	Exon	A	T	0.79	0.79	0.77	0.82	0.87	0.016	0.60				
rs28362677	*BTNL2*	Missense	G	A	0.87	0.82	0.84	0.90	0.78	0.002	1.92				
rs2076530	*BTNL2*	Missense	A	G	0.68	0.71	0.65	0.71	0.61	0.040	1.41				
rs3763313	*BTNL2*	Upstream-variant-2KB	A	C	0.79	0.82	0.82	0.78	0.70	0.008	1.63				
rs5007259	*BTNL2*	Promoter	T	C	0.59	0.68	0.63	0.56	0.50	0.024	1.43	0.035	2.14		
rs9268528	*BTNL2*	Promoter	A	G	0.66	0.76	0.73	0.60	0.71					0.014	1.78
rs3135351	*BTNL2/DRA*		T	G	0.24	0.26	0.30	0.19	0.22					0.016	1.85
rs3129843	*BTNL2/DRA*		G	A	0.13	0.16	0.18	0.08	0.10					0.004	2.63
rs9268644	*HLA-DRA*	Intron-variant	A	C	0.55	0.66	0.63	0.48	0.56					0.004	1.89
rs3129877	*HLA-DRA*	Intron-variant	A	G	0.32	0.34	0.38	0.26	0.38					0.023	1.71
rs3135392	*HLA-DRA*	Intron-variant	T	G	0.47	0.47	0.50	0.44	0.56	0.016	0.68				
rs3177928	*HLA-DRA*	utr-variant-3-prime	G	A	0.91	0.92	0.92	0.91	0.83	0.001	2.17				
rs6937545	*HLA-DRA*	Downstream variant	A	C	0.46	0.61	0.54	0.39	0.39			0.010	2.43	0.004	1.89

*NL, non-Löfgren sarcoidosis patient; LS, Sarcoidosis patient with Lofgren syndrome; NLR, non-Löfgren with resolved disease; NLP, non-Löfgren with persistent disease; n, number of samples*.

*The P-values are uncorrected*.

We did assays designing with AssayDesign software and performed the multiplex PCR and the iPLEX reaction using 9–10 ng of DNA as a template. The SNP allele separation based on the differences of the single base extension products used the Sequenom MassArray iPLEX system (Sequenom, San Diego, CA, USA). The *HLA-DRB1* genotypes for the samples were previously published (10, 12, 18) including genotypes for 497 cases and 517 controls.

### Statistical Analysis

We compared allele frequencies of 89 SNPs of the discovery sample and of 16 SNPs of the replication samples between different groups (NL vs. controls, LS vs. controls, NL resolved vs. NL persistent) by a case-control association analysis (Chi-square χ^2^ test, PLINK software) (21) (Table S2 in Supplementary Material). We applied the following quality control filters: minimum call rate per sample of 90%, SNP MAF >0.01, and Hardy–Weinberg equilibrium >0.001. Total success rate for accepted SNP arrays was 99% in the discovery sample and 98% in the replication samples together.

We combined the results from SNP analyses of the discovery and replication samples for a joint analysis using the random effects model (PLINK software). We used the Benjamini–Hochberg False Discovery Rate method for correction for multiple testing. SNP associations and *HLA-DRB1* alleles were adjusted for sex, and all the *HLA-DRB1* alleles by a logistic regression [PAWStatistics 18.0, PLINK software (21)].

To determine the genetic relationships among individuals belonging to four different European populations (Finnish, Swedish, Dutch, and Czech), we performed principal coordinates analyses (PCoA), a multivariate technique, using pairwise individual-by-individual linear genetic distance matrix with covariance standardized method in GenAlEx 6.5 tool. This linear genetic distance approach correlates genetic and geographic distances (Mantel test) within the dataset of 16 SNPs in healthy control subjects and sarcoidosis patients across the studied European populations. The method identified the major variation axis within our multidimensional genotype data set. As each successive axis explained proportionately less of the total genetic variation, the first two axes were used to reveal the major separation among individuals. Heterogeneity between the studies was evaluated using the *I*^2^ metric with a heterogeneity threshold of *I*^2^ < 25% (low heterogeneity) (22). The haplotypes were constructed and pairwise LD (*r*^2^) detected using SNP Haploview software (23). The LD structure in control samples from all study populations was compared to the LD structure of population genotype data originating from Phase 3 of the 1,000 Genomes Project (24). We estimated *HLA-DRB1-SNP* haplotype frequencies from allele data using the Bayesian method with PHASE v. 2.1.1 (23). The *HLA-DRB1* low-resolution data (HLA-DRB1*01, *03, *04, *07 *08, *10, *11, *12, *13, *14, *15, *16) was available for the Finnish (*n* = 187 cases, *n* = 150 controls), and for a part of the Swedish (*n* = 184 cases, *n* = 187 controls) and Czech (*n* = 90 cases, *n* = 180 controls) samples. The HapMap3 proxy SNPs (*r*^2^ > 0.8) were detected using SNAP software (25, 26). We followed the STREIS principles in immunogenomic data analysis (27).

### Analysis of Expression Quantitative Trait Loci (eQTL) Data and Pathway Connectivity Analysis

To further investigate the possibly functional effects of the significant SNPs, we used GENe Expression Variation (GeneVar) (28) database to study the eQTL.

## Results

A total of 805 sarcoidosis patients and 870 controls were included in this study. The discovery sample set consisted of Finnish sarcoidosis patients and controls. Swedish, Dutch, and Czech data sets were the replication samples and used for the joint analysis. We analyzed 89 SNPs for the discovery samples and selected 16 of these SNPs for the replication and the joint analysis.

### Thirteen SNPs Showed Association with Sarcoidosis in the Discovery Sample

Seven SNPs showed association (uncorrected *P* < 0.05) with NL in the discovery sample (Table 2). The strongest association with NL was observed for rs3177928 (*P* = 0.001, OR = 2.17) located within downstream of *HLA-DRA*. Four SNPs in *BTNL2* showed significant association with NL, of these, the most significant rs28362677 (*P* = 0.002, OR = 1.92) was a missense variant (Ser360Gly). Two SNPs (rs5007259 in *BTNL2* promoter and rs6937545 in downstream of *HLA-DRA*) associated with LS. Six SNPs [rs9268528 (*BTNL2* promoter), rs3135351, and rs3129843 (between genes *HLA-DRA* and *BTNL2*), rs9268644 and rs3129877 (*HLA-DRA* intronic), rs6937545 (downstream of *HLA-DRA*)] were associated with the disease course of sarcoidosis (Table 2). Neither *TNF* nor *LTA* variants were significant in the discovery sample or showed strong LD with other MHC variants, hence, not included in the replication stage. For discovery stage, detailed results of the allele frequencies from 89 SNPs are found in Table S1 in Supplementary Material.

### Shared and Population-Specific SNP Associations with Sarcoidosis in the Replication Samples

Heterogeneity at association level was observed among the samples and *BTNL2* SNPs showed population-specific differences (Table S2 in Supplementary Material). The sarcoidosis-associated *BTNL2* missense SNP rs2076530 (16) and *BTNL2* promoter SNP rs5007259 were associated in Swedish (rs2076530 with borderline association) and Czech samples (NL vs. controls), but were not replicated in the Dutch sample. In Dutch samples, the most significant exonic *BTNL2* SNP rs28362677 (NL 93% vs. controls 83%; *P* = 0.00016, OR = 2.5) was not replicated among Swedish and Czech samples.

### PCoA for Population Stratification

Due to different European origins, the samples were checked for ancestral homogeneity using the PCoA. In all study populations, the healthy control subjects seemed to be more diverse than the sarcoidosis patients. The Finnish and Czech healthy controls were most diverged, while Czech and Swedish sarcoidosis patients were among least diverged. Overall, a homogenous clustering with absence of any major cluster(s) among the individuals and low percent of variations was determined (Figures S1 and S2 in Supplementary Material).

### Joint Analysis Combining Results from Discovery and Replication Samples

The allelic associations found in the discovery and replication samples (after filtering, 765 sarcoidosis patients and 859 controls) were used for the joint analysis. Four of the SNPs were replicated in all four populations: rs3763313 (NL vs. C; *BTNL2* upstream-variant), rs3177928 (NL vs. C; *HLA-DRA* downstream variant), rs5007259 (L vs. C; *BTNL2* upstream-variant), and rs6937545 (L vs. C; *HLA-DRA* downstream variant).

In the joint analysis, all the other SNPs (*P*-value for random effects < 0.05), except rs9268528, were associated with at least one of the disease phenotypes (*I*^2^ < 25%) (Table S2 in Supplementary Material). Seven SNPs in *AGER, BTNL2*, and *HLA-DRA* associated with NL, two most significant being downstream variant of *HLA-DRA* (rs3177928, *P* = 0.0000002, OR = 1.9) and *BTNL2* promoter SNP (rs3763313, *P* = 0.000001, OR = 1.6). Eight SNPs associated with LS (Table S2 in Supplementary Material). Three of these SNPs had *P* < 10^−8^: rs3130349 in *AGER* (OR = 0.39), rs3129843 between genes *BTNL2* and *HLA-DRA* (OR = 3.4), and rs6937545 in *HLA-DRA* (OR = 2.3). Fifty-seven percent (78/136) of LS patients were Swedish. Five SNPs were associated with the disease course of sarcoidosis (*BTNL2* exonic SNP rs28362677, *BTNL2* promoter SNP rs5007259, SNPs rs3135351 and rs3129843 between genes *BTNL2* and *HLA-DRA*, and *HLA-DRA* downstream SNP rs3129877) (Table S2 in Supplementary Material). All the associations, except rs5007259 in NLR vs. NLP, remained significant after correcting for multiple comparisons (Table S2 in Supplementary Material).

### Haplotype Analysis Showed Population-Specific Structures

**Figure 1:**
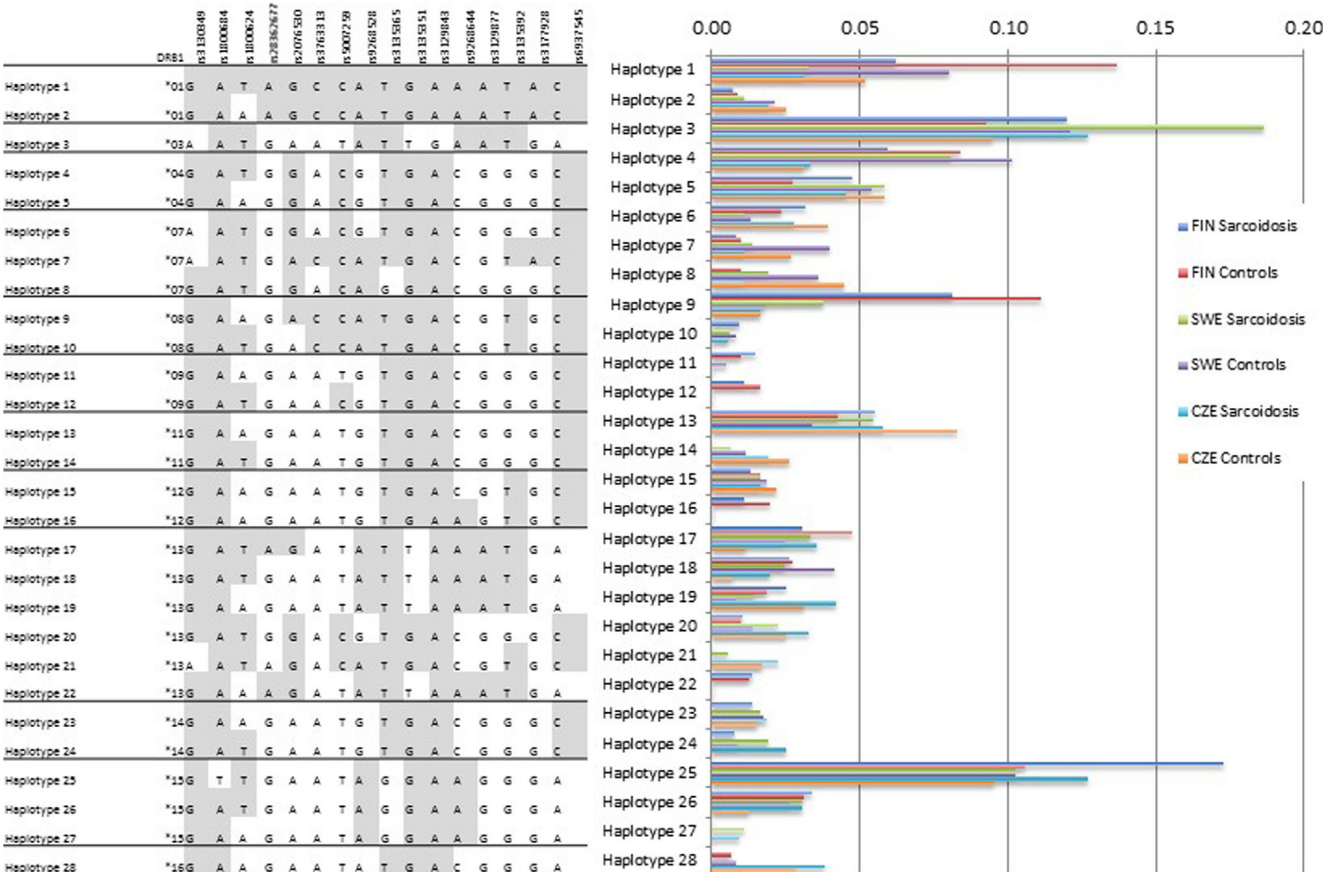
***HLA-DRB1*-SNP haplotype frequencies**. *HLA-DRB1* data were available for Finnish, Swedish, and Czech samples. Subjects with missing SNP genotype were excluded from the analysis. SNP order is the same as in the Table 2. The analysis shows population-specific differences in haplotype structures. Haplotype frequencies (2*n*) > 0.01 are presented.

Haplotype analysis was concordant with the single allele findings showing that population-specific structures occur (Figure 1). Haplotype 1 with HLA-DRB1*01 was more common in the Finnish sample (cases 6%, controls 14%) than in the Swedish (cases 3%, controls 8%) or the Czech sample (cases 3%, controls 5%). The haplotype 3 with HLA-DRB1*03 was the most frequent haplotype in the Swedish sample (cases 19%, controls 12%) probably due to the high number of LS patients that commonly share the allele. Haplotype 28 with HLA-DRB1*16 was missing or rare in the Finnish and Swedish sample.

### *HLA-DRB1*-Independent Association in Four SNPs

Addressing the question of whether the SNP associations were secondary to the *HLA-DRB1* associations found previously (10, 12, 18, 29), we performed logistic regression adjusting for the low-resolution *HLA-DRB1* alleles (Table 3). In the comparisons of “NL vs. controls (recessive model),” SNPs rs3135365 and rs3177928, HLA-DRB1*15 and *16 were suggested as independent variants (*P* = 0.015, OR = 2.15, CI 95% = 1.16–3.99; *P* < 0.001, OR = 2.19, CI 95% = 1.54–3.15; *P* < 0.001, OR = 3.49, CI 95% = 1.87–6.53, *P* = 0.044, OR = 0.43, CI 95% = 0.19–0.98; respectively). For LS using a dominant model, the downstream SNP of *HLA-DRA* (rs6937545; *P* = 0.012, OR = 3.49, CI 95% = 1.32–9.25) and HLA-DRB1*03 (*P* < 0.001, OR = 4.67, CI 95% = 2.18–10.12), *13 (*P* = 0.034, OR = 2.41, CI 95% = 1.07–5.42), and *14 (*P* = 0.007, OR = 5.38, CI 95% = 1.59–18.2) alleles were suggested as significant predisposing markers. In the analysis of NLR vs. NLP (recessive model), no independent associations were detected in studied SNPs. None of the *AGER* SNPs remained significant after adjusting for *HLA-DRB1* alleles showing strong LD with *HLA-DRB1*.

**Table 3:** **Disease associated SNPs in the major histocompatibility complex region in the joint analysis (Finnish, Swedish, Dutch, and Czech) and associations after adjusting for *HLA-DRB1* low-resolution alleles**.

		Unadjusted		Adjusted for *HLA-DRB1*	
SNP	Location	*P*	OR	*P*	OR
**NL vs. C**					
Rs1800684	*AGER* (coding)	0.003	0.727		
Rs2076530	*BTNL2* (coding)	<0.001	1.339		
rs3763313	*BTNL2* (5′)	<0.001	1.633		
rs3135365		0.009	0.777	0.015	2.15
rs3129877	*HLA-DRA* (intronic)	0.020	0.819		
rs3135392	*HLA-DRA* (intronic)	0.0058	0.805	<0.001	2.19
rs3177928	*HLA-DRA* (3′)	<0.001	1.898		
				^a^HLA-DRB1*15/*16	
**LS vs. C**					
rs3130349	*RNF5* (coding)	<0.001	0.3925		
rs1800624	*PBX2* (3′)	0.00125	1.6955		
rs2076530	*BTNL2* (coding)	0.008553	1.4486		
rs3763313	*BTNL2* (5′)	<0.001	2.0534		
rs5007259	*BTNL2* (5′)	<0.001	1.9936		
rs3129843		<0.001	3.4443		
rs9268644	*HLA-DRA* (intronic)	0.0009935	1.6901		
rs6937545		<0.001	2.2837	0.012	3.49
				^a^HLA-DRB1*03/*13/*14	
**NLR vs. NLP**					
rs28362677	*BTNL2* (coding)	0.004712	0.5743		
rs3135351		0.003817	1.5283		
rs3129843		0.002962	1.8574		
rs3129877	*HLA-DRA* (intronic)	0.004781	1.4506		

*^a^Associated HLA-DRB1 allele(s)*.

*NL, non-Löfgren sarcoidosis patient; LS, Sarcoidosis patient with LS; NLR, non-Löfgren with resolved disease; NLP, non-Löfgren with persistent disease; C, controls*.

### LD Structure Confirmed SNP Selection Strategy

**Figure 2:**
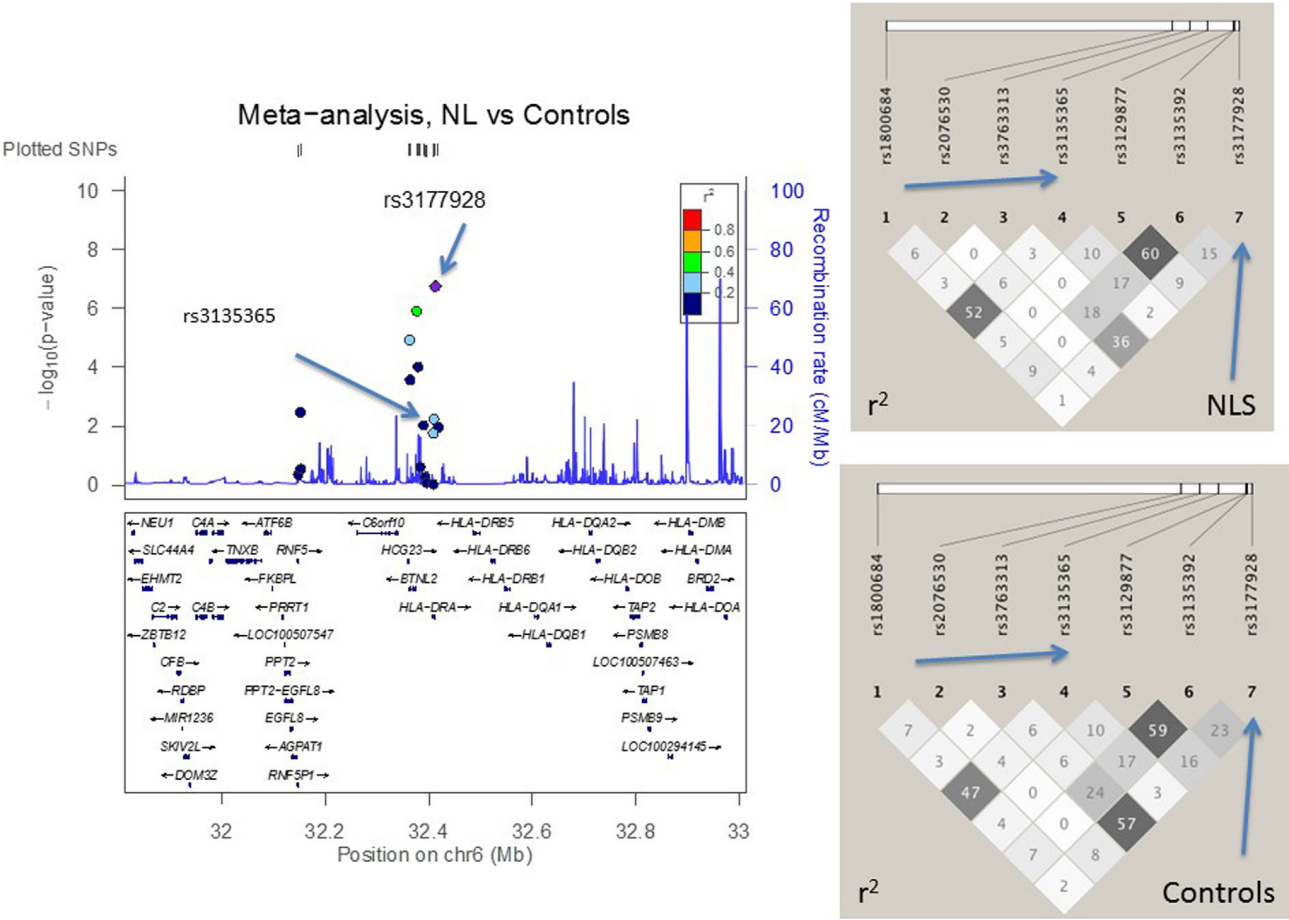
**The meta-analysis of non-Löfgren sarcoidosis (NL) vs. controls with Finnish, Swedish, Dutch, and Czech samples**. The linkage disequilibrium (LD) information (*r*^2^) is shown by color-coded dots and the recombination rates where the peaks are the hotspots are based on the HapMap data. The LD structure (*r*^2^) in the current study for the top significant SNPs (*I*^2^ < 25) are shown on the right. After adjusting for *HLA-DRB1* alleles, the SNPs that remained significant are pointed with an arrow.

**Figure 3:**
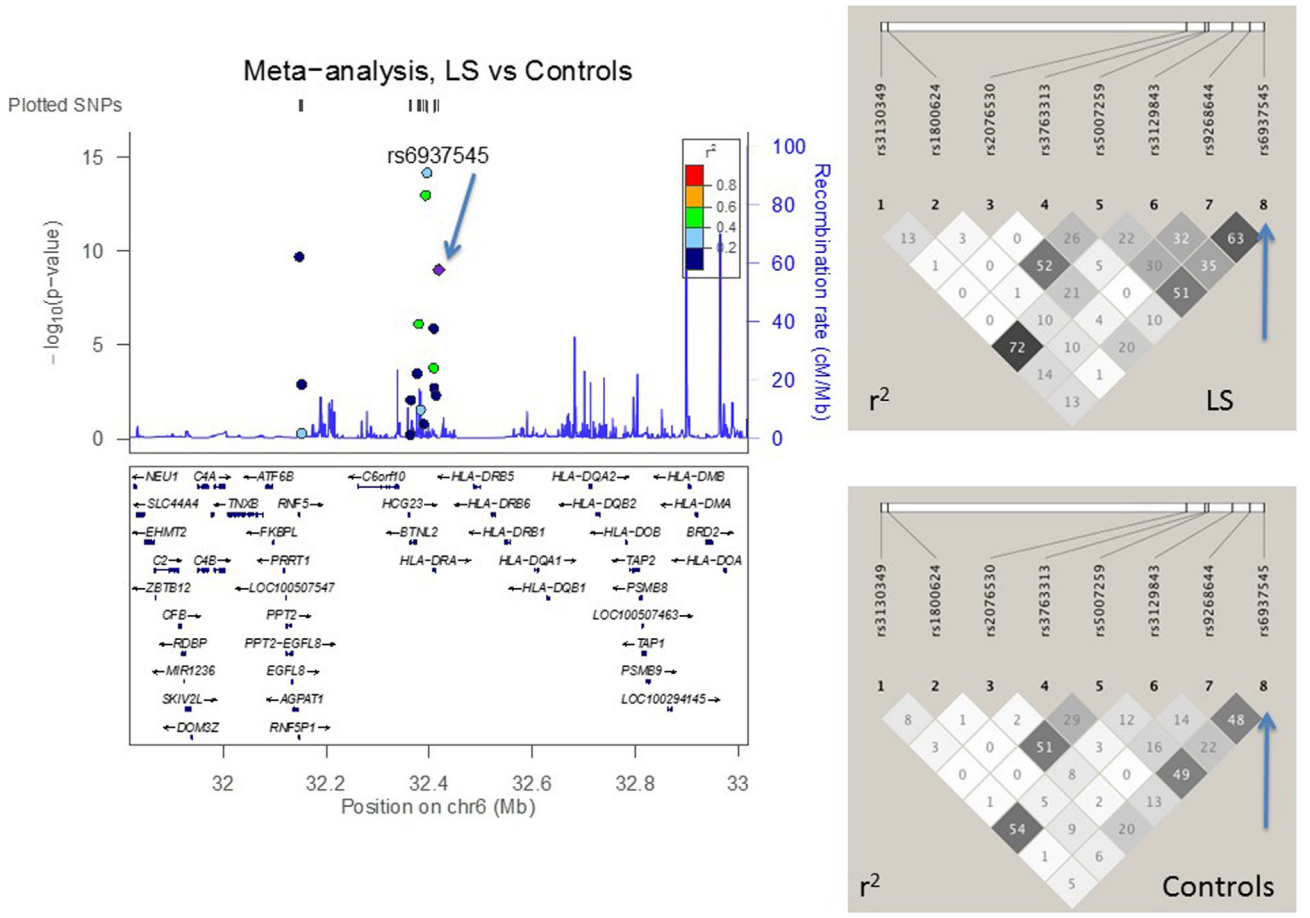
**The meta-analysis of Löfgren (LS) vs. controls with Finnish, Swedish, and Czech samples**. The linkage disequilibrium (LD) information (*r*^2^) is shown by color-coded dots and the recombination rates where the peaks are the hotspots are based on the HapMap data. The LD structure (*r*^2^) in the current study for the top significant SNPs (*I*^2^ < 25) are shown on the right. After adjusting for *HLA-DRB1* alleles, the SNPs that remained significant are pointed with an arrow.

**Figure 4:**
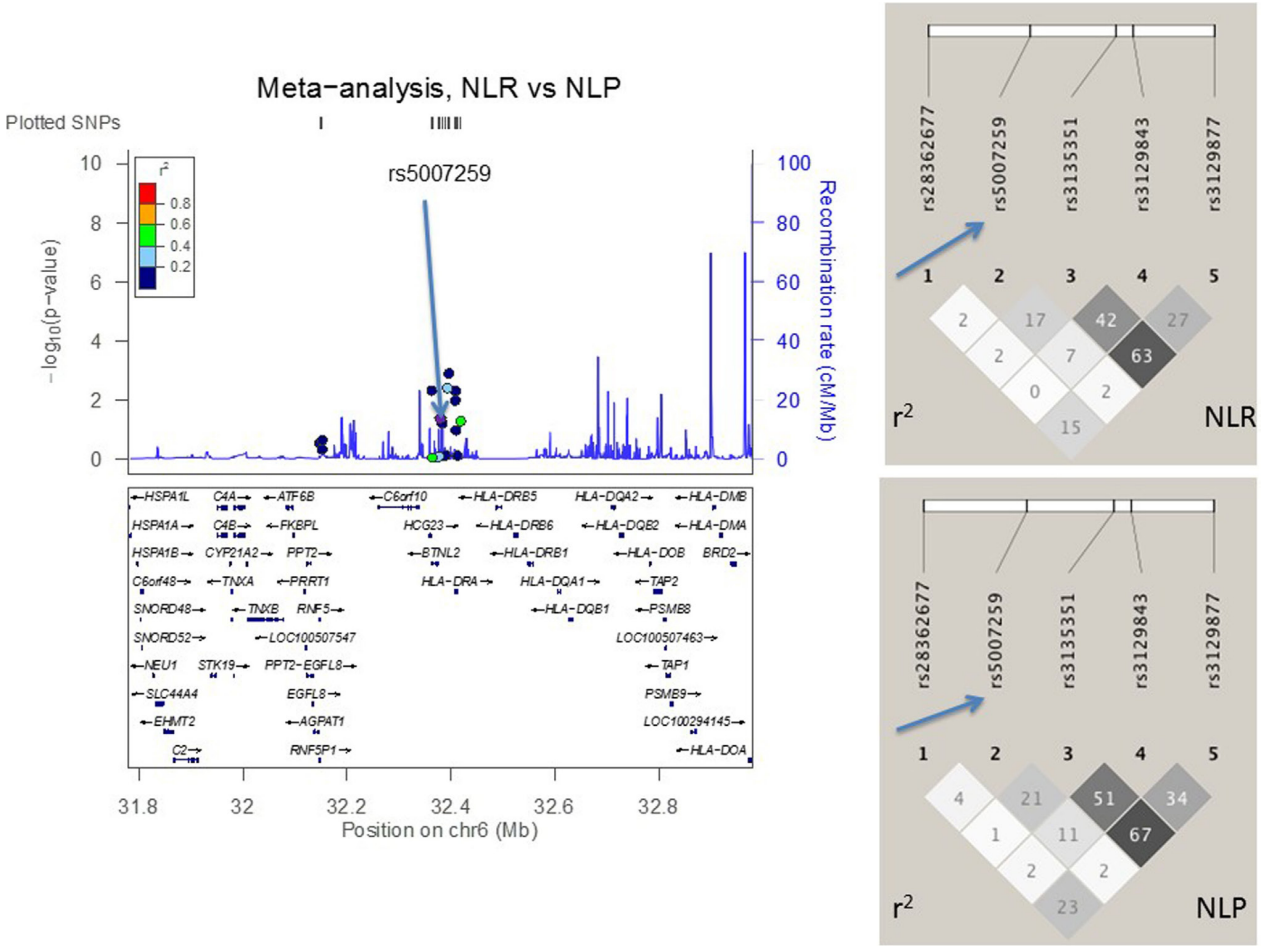
**The meta-analysis of sarcoidosis subgroups (resolved sarcoidosis, NLR vs. persistent sarcoidosis, NLP) with Finnish, Swedish, Dutch, and Czech samples**. The linkage disequilibrium (LD) information (*r*^2^) is shown by color-coded dots and the recombination rates where the peaks are the hotspots are based on the HapMap data. The LD structure (*r*^2^) in the current study for the top significant SNPs (*I*^2^ < 25) are shown on the right. After adjusting for *HLA-DRB1* alleles, the SNPs that remained significant are pointed with an arrow.

Figures 2–4 visualize genotyped SNPs (*P* < 0.05 and *I*^2^ < 25%) and the LD structure (*r*^2^) based on HapMap data for both cases and controls. Estimated recombination rates are plotted to show the LD structure around the associated SNPs. Gene annotations were adapted from the University of California at Santa Cruz Genome Browser.^2^ As none of the genotyped SNPs had strong (*r*^2^ > 0.8) pairwise LD in this study, it confirmed the selection strategy where only one SNP from each LD block was genotyped. LD structure of control populations corresponded to the 1,000 Genomes population data (data not shown).

### Four SNPs Act As Local eQTL for *HLA-DRB1/-DRB5*

Using the GeneVar database (28), we analyzed the available eQTL-SNP-gene data to identify eQTL genes surrounding independent SNPs [lymphoblastoid cell lines, CEU (30)]. The analysis suggested SNPs rs3135365, rs3177928, and rs6937545 act as cis-acting eQTL for *HLA-DRB1*, and rs3135365, rs6937545, and rs5007259 act as eQTL for *HLA-DRB5*. Figure S3 in Supplementary Material presents the SNP-probe association plots.

## Discussion

Major histocompatibility complex region contains immune mediator genes that have showed either predisposing or protective effects to sarcoidosis (12, 15). Here, we report a set of 16 SNPs located in the MHCs that are associated with sarcoidosis and shared between four European populations with distinct ancestral origins (Finnish, Swedish, Dutch, and Czech). Only two of these SNPs have been previously associated with sarcoidosis. After conditioning with *HLA-DRB1* alleles, three of the 16 SNPs remained independently associated with sarcoidosis.

We identified two novel *HLA-DRA* downstream variants that were independent of *HLA-DRB1* alleles: rs3177928 associated with NL and rs6937545 associated with LS. In a high-density genetic mapping study of extended MHC region in four European populations and one black African descent population, NL sarcoidosis showed similar association pattern: main associations were found in variants located in the MHC class II region (31). MHC II region associations were found in LS patients as well. Recent non-sarcoidosis studies showed that rs3177928 was associated with lipoprotein metabolism (32) and connected with inflammatory mechanisms (33–35). *HLA-DRA* encodes the α-subunit of the *HLA-DR* cell surface receptor and expresses on antigen-presenting cells. *HLA-DRA* shows strong LD structure and its variants regulate the expression of other MHC genes (36). Both rs3177928 and rs6937545 are also cis-acting eQTL affecting the expression of *HLA-DRB1* and are not in LD with other *HLA-DRA* gene variants previously associated with sarcoidosis (6–8). Interestingly, *HLA-DRA* has shown to have SNP–SNP (8) interaction with *ANXA11*, another sarcoidosis associated gene. *ANXA11* has an essential role in cell division and apoptosis (6, 7).

The increasing line of evidence suggests that *BTNL2*, a member of the immunoglobulin gene superfamily, is a key element for sarcoidosis predisposition (6, 15, 16, 37). *BTNL2* regulates T cells and is expressed on dendritic cells (15, 38). Here, the novel variant in *BTNL2* 5’ region associated independently with distinct sarcoidosis phenotype (rs3135365 with NL) supporting the importance of the *BTNL2* promoter region in sarcoidosis disease development. Several other *BTNL2* SNPs also reached the level of significance. However, they showed either heterogeneity between populations (e.g., exonic SNP rs28362677 and 5’ upstream SNP rs5007259) or the association weakened after adjustments with *HLA-DRB1*, as occurred with the splice-site variant of *BTNL2* (rs2076530) that was previously associated with chronic sarcoidosis in subjects of European descent (6, 16, 30, 37, 39–41). The strong LD between *BTNL2* and *HLA-DRB1* complicates the interpretation of the results and the inconsistency with previous studies may be due to differences in the study design (heterogeneity in diagnosis) and population origin (16, 42, 43). Given that, we suggest that district variants of *BTNL2* are present only in certain populations, like exonic SNP rs28362677 in the Dutch sample that may explain the lack of replication of rs2076530 (11, 40). Fine-mapping studies of *BTNL2* in large multi-population sample would shed light on the allelic heterogeneity of *BTNL2*.

HLA allele variation in Europe follows the North to Southeast axis and as expected, different HLA haplotype profiles were observed in this study (44). We noticed that the use of different population data was advantageous for the *HLA-DRB1* adjustments, because the populations have different chromosomal regions where LD breaks down as well as distinct allele frequencies (4). HLA-DRB1*03 has been associated with sarcoidosis with favorable prognosis and Löfgren’s syndrome in several populations with European descent, especially in Scandinavian countries (10, 39, 45–47). In Japan, the HLA-DRB1*03 allele is rare, explaining the non-association of the variant with sarcoidosis (48). We showed that LS-associated SNPs were inherited as a conserved block with the HLA-DRB1*03, especially in the Swedish sample (10), and after conditioning with *HLA-DRB1* alleles, only one SNP remained significant (rs6937545 in *HLA-DRA* downstream). Given the strong LD within MHC genes, we highlight the importance to use of HLA genotypes in addition to SNP data when aiming to pinpoint the causal variants within MHC region.

There are several strengths in our study. The sample size in the joint analysis of the discovery and replication sets was relatively large for case-control study in the context of sarcoidosis and its incidence. Our fine-mapping strategy of MHC candidate genes in different ancestral origins aimed to detect variants associated with different disease phenotypes shared in different populations. Previous studies have pointed out that the precise clinical characterization of the patients is essential, because sarcoidosis is a highly heterogeneous disease. To overcome the disease heterogeneity, we subdivided the patients according to clinical phenotypes (49, 50).

In conclusion, we found novel SNPs in *BTNL2* and *HLA-DRA* regions associating with sarcoidosis. Our finding further establishes that polymorphisms in the *HLA-DRA* and *BTNL2* have a role in sarcoidosis susceptibility. This multi-population study demonstrates that at least a part of these associations are *HLA-DRB1* independent (e.g., not due to LD), and shared across ancestral origins. The variants that were independent of *HLA-DRB1* associations, acted as eQTL for *HLA-DRB1* and/or *-DRB5*, suggesting a role in regulating gene expression. Future functional studies with larger sample are required to reveal the causal regulatory variation at this locus and the immunogenetic basis related to sarcoidosis.

## Ethics Statement

The study was performed with approval of institutional ethical committees at respective centers (Ethics Commitee of the Department of Internal Medicine, Hospital District of Helsinki and Uusimaa, Finland; Ethics Committee of the University Hospital and Medical Faculty of Palacky University, Olomouc, Czech Republic; Ethics Committee of the Karolinska University Hospital, Solna, Sweden; Ethics Committee the University Medical Center Utrecht, Netherlands).

## Author Contributions

M-LL, OS, AP, MP, AW, and EL conceived and designed the work. JG, CM, MP, AE, JG, VK, FM, LP, AP, MR, MS, M-LL, and OS contributed to data acquisition. AW and EL gathered SNP information and did all the analyses and interpretation. AK and VA assisted with data analyzing. AW and EL drafted the manuscript after its revision for important intellectual context by OS, AP, MP, VA, FM, AK, M-LL, CM, AE, JG, and MR. AW and EL finalized the article. All authors have read and approved the final manuscript and agreed to be accountable for all aspects of the work.

## Conflict of Interest Statement

The authors declare that the research was conducted in the absence of any commercial or financial relationships that could be construed as a potential conflict of interest.

## Footnotes

^1^http://www.ncbi.nlm.nih.gov/projects/SNP.

^2^http://genome.ucsc.edu/.
