# Supplementary material for: SNP Variants in Major Histocompatibility Complex Are Associated with Sarcoidosis Susceptibility—A Joint Analysis in Four European Populations
Source: Front Immunol. 2017 Apr 19;8:422. doi: 10.3389/fimmu.2017.00422 (PMC5395694; doi:10.3389/fimmu.2017.00422)
Supplement: Supplementary file 1 [file Table_1.pdf]

Supplementary Table S1

Alltogether 89 SNPs were chosen within and outside of LTA, TNFalfa, AGER, BTNL2 and HLA-DRA genes in chromosome 6, and genotyped in the discovery sample set (F

SNP	other name	Closest Gene	Base Pair position (hg18)	Alleles A1/A2 (dbSNP)	MAF (ESP)	MAF (1000g EUR)
rs2009658		LTA (5upstream)	31538244	C/G	-	0.1478
rs2239704		LTA (5upstream)	31540141	A/C	-	0.3813
rs2229094		LTA (coding)	31540556	T/C	0.270395	0.3021
rs2229092		LTA (coding)	31540757	A/C	0.070137	0.062
rs1041981		LTA (coding)	31540784	C/A	0.330934	0.3166
rs1799964	TNF -1031	LTA (3downstream)	31542308	T/C	-	0.215
rs1799724	TNF -857 or -850	LTA (3downstream)	31542482	C/T	-	0.0805
rs1800629	TNF -308	LTA (3downstream)	31543031	G/A	0.150609	0.1359
rs361525	TNF -238	LTA (3downstream)	31543101	G/A	-	0.066
rs3179060		TNF (coding)	31543672	C/A	-	-
rs35131721		TNF (coding)	31544315	C/T	-	-
rs4645843		TNF (coding)	31544562	C/T	0.002953	-
rs1800620		TNF (coding)	31544591	G/A	-	-
rs3093664		TNF (intronic)	31544642	A/G	0.077335	0.1016
rs1800618		TNF (coding)	31544918	C/T	-	-
rs11574936		TNF (coding)	31545193	T/A	0.000369	-
rs769178		LTB (3downstream)	31547514	G/T	-	0.0752
rs4647186		LTB (coding)	31549364	G/C	-	-
rs3093554		LTB (coding)	31549407	C/T	0	0
rs3093553		LTB (non-coding)	31549556	T/G	0.064784	0.083
rs2256965		LST1 (non-coding)	31555130	A/G/T	0.423372	0.4288
rs4947332		SLC44A4 (intronic)	31834197	C/T	-	0.033
rs3134945		AGPAT1 (5upstream)	32146492	C/A	-	0.195
**rs3130349**		**RNF5 (coding)**	**32147696**	**G/A**	0.182326	0.164
rs17846806		AGER (coding)	32151346	C/T	-	-
rs35795092		AGER (coding)	32151420	G/C	0.053565	0.051
rs2070600		AGER (coding)	32151443	C/T	0.045622	0.053
rs35802968		AGER (coding)	32151664	C/T	0.000185	-
rs35030981		AGER (coding)	32151674	C/T	0	-
rs17846804		AGER (coding)	32151734	G/A	-	-
**rs1800684**		**AGER (coding)**	**32151994**	**A/T**	0.133813	-
**rs1800624**	**AGER-374**	**PBX2 (3downstream)**	**32152387**	**A/T**	-	-
rs9268473			32355683	A/G	-	-
**rs28362677**		**BTNL2 (coding)**	**32362741**	**C/T**	0.144302	-
rs28362678		BTNL2 (coding)	32362745	G/A	0.144302	0.157
**rs2076530**		**BTNL2 (coding)**	**32363816**	**T/C**	0.438354	0.434
rs9268480		BTNL2 (coding)	32363844	C/T	0.281758	0.27
rs2076529		BTNL2 (coding)	32363955	T/C	0.430786	-
rs3129954		BTNL2 (intronic)	32365580	A/G	-	-
rs3817962		BTNL2 (intronic)	32368314	C/A	-	0.268
rs3793127		BTNL2 (intronic)	32371915	C/T	-	0.213
rs28362682		BTNL2 (coding)	32372863	A/T	0.078996	0.103
rs28362683		BTNL2 (coding)	32372963	G/A	0.078996	-
rs3763311		BTNL2 (5upstream)	32376176	C/T	-	0.286
rs3763312		BTNL2 (5upstream)	32376348	G/A	-	0.213
**rs3763313**		**BTNL2 (5upstream)**	**32376471**	**A/C**	-	0.245
rs3763315		BTNL2 (5upstream)	32376654	G/T	-	0.003
rs3763317		BTNL2 (5upstream)	32376788	C/T	-	-
**rs5007259**		**BTNL2 (5upstream)**	**32379101**	**T/C**	-	-
rs17208888		BTNL2 (5upstream)	32379506	G/A	-	-
rs9405098		BTNL2 (5upstream)	32379736	G/A	-	-
**rs9268528**			**32383108**	**G/A**	-	0.372
rs9268541			32384527	C/T	-	-
rs2395166			32388275	C/T	-	-
**rs3135365**			**32389255**	**C/A**	-	0.182
rs9268560			32389512	C/G	-	0.447
rs3135363			32389648	A/G	-	-
**rs3135351**			**32392945**	**C/A**	-	0.137
rs3135344			32395036	C/T	-	0.325
**rs3129843**			**32395726**	**A/G**	-	0.079
rs3135341			32398748	T/G/C/A	-	0.197
rs16870148			32399636	G/A	-	0.009
rs2027856			32402705	C/T/G/A	-	-
rs3129871		HLA-DRA (5upstream)	32406342	A/C	-	-
rs9405035		HLA-DRA (5upstream)	32407068	G/A	-	-
**rs9268644**		**HLA-DRA (intronic)**	**32408044**	**A/C**	-	0.403
**rs3129877**		**HLA-DRA (intronic)**	**32408597**	**G/A**	-	-
**rs3135392**		**HLA-DRA (intronic)**	**32409242**	**C/A**	-	-
rs3129882		HLA-DRA (intronic)	32409530	G/A	-	0.453
rs8084		HLA-DRA (coding)	32411035	A/C	0.430786	-
rs2239804		HLA-DRA (intronic)	32411523	T/C	0.463257	0.436
rs11544315		HLA-DRA (coding)	32411573	C/T	0.038391	-
**rs3177928**		**HLA-DRA (3downstream)**	**32412435**	**G/A**	-	-
rs3135388		HLA-DRA (3downstream)	32413051	A/G	-	-
rs2213585		HLA-DRA (3downstream)	32413150	G/A	-	-
**rs6937545**			**32418031**	**C/A**	-	-
rs9268833		HLA-DRB9 (non-coding)	32428062	T/C	-	-
rs36056234		HLA-DRB9 (non-coding)	32428917	G/A	-	0.016
rs6919855		HLA-DRB9 (non-coding)	32429011	C/T	-	-
rs7766843		HLA-DRB9 (non-coding)	32430729	C/T	-	0.255
rs2395185		HLA-DRB9 (non-coding)	32433167	G/T	-	-
rs9268979		HLA-DRB9 (non-coding)	32435044	C/T	-	-
rs9405040		HLA-DRB9 (non-coding)	32439393	A/C	-	0.276
rs7748472			32448763	A/G	-	0.041
rs13198498			32452033	G/C	-	-
rs28760027			32509985	G/A	-	0.1504
rs34369284			32514144	G/A	-	0.363
rs28594633		HLA-DRB6 (non-coding)	32524224	G/A	-	0.022
rs35464393			32530198	T/C	-	0.258
