# Supplementary material for: SNP Variants in Major Histocompatibility Complex Are Associated with Sarcoidosis Susceptibility—A Joint Analysis in Four European Populations
Source: Front Immunol. 2017 Apr 19;8:422. doi: 10.3389/fimmu.2017.00422 (PMC5395694; doi:10.3389/fimmu.2017.00422)
Supplement: Supplementary file 2 [file Table_2.pdf]

Supplementary Table S2

Association analysis in the Finnish discovery and replication samples (Dutch, Swedish, Czech) and the joint analysis combining the discovery and replication sample sets. The SNPs with P value < 0.05 are bolded.
The significant SNPs with low heterogeneity (I2 < 25%) are shown in grey.

							Replication sample														
			Finnish				Swedish				Dutch				Czech				Joint analysis		
SNP	A1	A2	P	OR	L95	U95	P	OR	L95	U95	P	OR	L95	U95	P	OR	L95	U95	P	OR	I
NL vs C			NL(n=168) vs C (n=150)				NL(n=112) vs C (n=358)				NL(n=90) vs C (n=173)				NL(n=169) vs C (n=178)				NL(n=629) vs C (n=859)		
rs3130349	G	A	0.284	0.786	0.5058	1.221	0.1016	1.436	0.9295	2.218	0.5873	1.11	0.7611	1.619	0.7372	1.067	0.732	1.554	0.509	1.078	18.23
**rs1800684**	**A**	**T**	**0.016**	**0.5953**	**0.3891**	**0.9109**	0.1145	0.7129	0.4676	1.087	0.6758	0.9149	0.603	1.388	0.1328	0.7123	0.457	1.11	**0.003***	**0.727**	**0**
rs1800624	T	A	0.670	0.9305	0.6683	1.296	0.07818	0.7472	0.5399	1.034	0.9788	0.9955	0.7121	1.392	0.9455	1.011	0.739	1.384	0.285	0.915	0
**rs28362677**	**G**	**A**	**0.002**	**1.922**	**1.262**	**2.927**	0.1849	1.368	0.8596	2.177	**0.0001563**	**2.484**	**1.532**	**4.026**	0.4551	1.184	0.76	1.843	**0.002**	**1.660**	**50.35**
**rs2076530**	**A**	**G**	**0.040**	**1.407**	**1.015**	**1.95**	0.05729	1.351	0.9902	1.844	0.2079	1.218	0.896	1.655	**0.03493**	**1.399**	**1.024**	**1.913**	**0.0003***	**1.339**	**0**
**rs3763313**	**A**	**C**	**0.008**	**1.632**	**1.136**	**2.345**	**0.02956**	**1.573**	**1.043**	**2.371**	**0.02766**	**1.542**	**1.047**	**2.27**	**0.006012**	**1.839**	**1.186**	**2.852**	**0.000001***	**1.633**	**0**
**rs5007259**	**T**	**C**	**0.024**	**1.433**	**1.047**	**1.962**	**0.01265**	**1.468**	**1.085**	**1.988**	0.8397	0.9699	0.721	1.305	**0.000805**	**1.682**	**1.24**	**2.282**	**0.011**	**1.358**	**58.35**
rs9268528	A	G	0.182	0.7954	0.5682	1.114	0.9733	0.9947	0.7306	1.354	0.2507	0.8361	0.6161	1.135	0.9078	1.018	0.753	1.377	0.245	0.912	0
rs3135365	T	G	0.085	0.7033	0.4709	1.05	0.06114	0.7064	0.4904	1.018	0.3698	0.8415	0.5769	1.227	0.4726	0.8703	0.596	1.272	**0.009***	**0.777**	**0**
rs3135351	T	G	0.474	1.145	0.7909	1.656	0.5828	0.8977	0.6108	1.319	0.5807	0.9044	0.6332	1.292	0.08462	1.419	0.952	2.113	0.566	1.063	18.98
rs3129843	G	A	0.214	1.371	0.8326	2.259	0.2343	0.7382	0.447	1.219	0.4393	0.8361	0.531	1.317	0.7248	1.095	0.661	1.812	0.853	0.975	17.08
rs9268644	A	C	0.878	0.9757	0.7134	1.335	0.8369	1.032	0.764	1.394	0.1	0.7803	0.5806	1.049	0.07208	1.322	0.975	1.792	0.942	1.008	49.94
rs3129877	A	G	0.104	0.7624	0.5496	1.057	0.1016	0.7556	0.5399	1.057	0.07022	0.7483	0.5465	1.025	0.6903	1.072	0.761	1.509	**0.02***	**0.819**	**3.28**
**rs3135392**	**T**	**G**	**0.016**	**0.6799**	**0.4971**	**0.9298**	0.149	0.7978	0.5869	1.085	0.1105	0.7841	0.5816	1.057	0.9523	0.9905	0.725	1.354	**0.0058***	**0.805**	**0**
**rs3177928**	**G**	**A**	**0.001**	**2.168**	**1.334**	**3.523**	**0.004588**	**2.1**	**1.245**	**3.541**	**0.01985**	**1.641**	**1.079**	**2.497**	**0.01984**	**1.845**	**1.095**	**3.108**	**0.0000002***	**1.898**	**0**
rs6937545	A	C	0.057	1.358	0.9901	1.864	0.08723	1.307	0.9612	1.778	0.422	0.8831	0.6519	1.196	**0.01959**	**1.442**	**1.06**	**1.962**	0.073	1.224	51.02
LS vs C			LS (n=19) vs C (n=150)				LS (n=78) vs C (n=358)				LS (n=0) vs C (n=0)				LS (n=39) vs C (n=178)				LS (n=136) vs C (n=859)		
rs3130349	G	A	0.199	0.5769	0.2471	1.347	**2.17E-08**	**0.3511**	**0.241**	**0.5116**	-	-	-	-	**0.001061**	**0.4209**	**0.248**	**0.7135**	**2.03E-10***	**0.3925**	**0**
rs1800684	A	T	0.199	0.5769	0.2471	1.347	0.3968	1.283	0.7199	2.287	-	-	-	-	**0.04788**	**3.165**	**0.953**	**10.51**	0.6233	1.2229	63.04
rs1800624	T	A	0.279	1.54	0.7016	3.379	**0.0248**	**1.651**	**1.062**	**2.567**	-	-	-	-	**0.03247**	**1.871**	**1.047**	**3.343**	**0.001***	**1.6955**	**0**
rs28362677	G	A	0.6135	1.249	0.5262	2.965	**0.06064**	**1.743**	**0.9693**	**3.134**	-	-	-	-	0.1497	0.6332	0.339	1.184	0.7533	1.1097	63.2
rs2076530	A	G	0.2144	1.591	0.7605	3.33	**0.01271**	**1.591**	**1.102**	**2.298**	-	-	-	-	0.5734	1.157	0.696	1.926	**0.009***	**1.4486**	**0**
rs3763313	A	C	0.1475	1.868	0.793	4.4	**0.001464**	**2.378**	**1.376**	**4.11**	-	-	-	-	0.1685	1.68	0.797	3.541	**0.0003***	**2.0534**	**0**
**rs5007259**	**T**	**C**	**0.03544**	**2.138**	**1.04**	**4.394**	**0.0001564**	**1.986**	**1.386**	**2.845**	-	-	-	-	**0.01157**	**1.938**	**1.153**	**3.256**	**7.63E-07***	**1.9936**	**0**
rs9268528	A	G	0.4936	1.316	0.5983	2.895	0.5801	1.107	0.7722	1.587	-	-	-	-	**0.002022**	**2.399**	**1.361**	**4.231**	0.1263	1.4868	60.8
rs3135365	T	G	0.05521	0.4792	0.223	1.03	0.06476	1.637	0.9661	2.775	-	-	-	-	**0.0114**	**3.2**	**1.243**	**8.238**	0.5668	1.3323	81.39
rs3135351	T	G	0.5483	1.266	0.5851	2.74	**8.35E-09**	**2.862**	**1.982**	**4.132**	-	-	-	-	**6.03E-09**	**4.517**	**2.644**	**7.718**	**0.0009**	**2.7033**	**71.68**
rs3129843	G	A	0.2432	1.752	0.6761	4.541	**7.74E-12**	**3.778**	**2.537**	**5.626**	-	-	-	-	**5.29E-06**	**3.845**	**2.09**	**7.073**	**3.44E-12***	**3.4443**	**11.54**
rs9268644	A	C	0.2353	1.532	0.7546	3.108	**0.04055**	**1.437**	**1.015**	**2.035**	-	-	-	-	**0.0006642**	**2.342**	**1.423**	**3.854**	**0.001***	**1.6901**	**21.33**
rs3129877	A	G	0.6495	0.8484	0.4173	1.725	**0.0008453**	**1.811**	**1.274**	**2.575**	-	-	-	-	**1.10E-06**	**3.375**	**2.036**	**5.594**	0.07674	1.8042	79.95
rs3135392	T	G	0.295	0.6976	0.3547	1.372	0.09121	1.348	0.9526	1.907	-	-	-	-	**1.26E-05**	**2.994**	**1.806**	**4.963**	0.3096	1.4494	83.94
rs3177928	G	A	0.1489	2.39	0.7076	8.069	**0.0006253**	**3.394**	**1.62**	**7.112**	-	-	-	-	0.841	1.081	0.504	2.319	0.06958	2.0293	55.98
**rs6937545**	**A**	**C**	**0.00988**	**2.432**	**1.219**	**4.853**	**6.30E-05**	**2.026**	**1.428**	**2.874**	-	-	-	-	**3.20E-05**	**2.827**	**1.712**	**4.67**	**1.00E-09***	**2.2837**	**0**
NLR vs NLP			NLR (n=79) vs NLP (n=89)				NLR (n=33) vs NLP (n=75)				NLR (n=90) vs NLP (n=90)				NLR (n=47) vs NLP (n=83)				NLR (n=249) vs NLP (n=337)		
rs3130349	G	A	0.3541	0.7609	0.4264	1.358	0.5253	0.7636	0.3317	1.758	0.2175	0.7119	0.4143	1.223	0.4221	1.299	0.685	2.464	0.2919	0.8456	0
rs1800684	A	T	0.2179	0.7168	0.4215	1.219	0.9727	1.014	0.4529	2.271	0.6603	1.138	0.6401	2.022	0.5487	0.8046	0.395	1.639	0.4595	0.888	0
rs1800624	T	A	0.07906	1.505	0.9526	2.378	0.1758	0.6593	0.3601	1.207	0.7198	1.09	0.6817	1.742	0.189	1.442	0.834	2.491	0.4216	1.15	43.04
rs28362677	G	A	0.05861	0.5361	0.279	1.03	0.8676	0.9267	0.3786	2.268	**0.02773**	**0.3941**	**0.1679**	**0.9254**	0.1777	0.6009	0.285	1.267	**0.005***	**0.5743**	**0**
rs2076530	A	G	0.2253	0.752	0.4742	1.193	0.3836	1.313	0.7108	2.427	0.5785	1.132	0.7317	1.75	0.7644	1.086	0.633	1.864	0.8765	1.02	0
rs3763313	A	C	0.3511	1.289	0.7552	2.201	0.6565	1.206	0.5273	2.76	0.2377	0.7041	0.3926	1.263	0.4345	0.7289	0.329	1.615	0.815	0.9619	0.02
rs5007259	T	C	0.2278	1.309	0.8449	2.028	0.1877	1.491	0.8218	2.704	0.2041	1.309	0.8636	1.984	0.7914	1.074	0.633	1.821	**0.04**	**1.2835**	**0**
**rs9268528**	A	G	**0.01434**	**1.775**	**1.119**	**2.815**	0.4229	0.7857	0.4354	1.418	0.5176	1.15	0.7532	1.755	0.2416	1.36	0.812	2.279	0.164	1.2482	38.05
rs3135365	T	G	0.2155	0.7204	0.4283	1.212	0.4867	1.287	0.6311	2.627	0.2381	1.364	0.8135	2.288	0.2187	0.6809	0.368	1.259	0.8057	0.9548	37.4
**rs3135351**	T	G	**0.01629**	**1.848**	**1.116**	**3.061**	0.2908	1.471	0.7171	3.016	0.1212	1.496	0.8973	2.495	0.5839	1.197	0.629	2.277	**0.004***	**1.5283**	**0**
**rs3129843**	G	A	**0.00407**	**2.633**	**1.336**	**5.189**	0.141	1.995	0.7846	5.074	0.04417	2.002	1.008	3.974	0.9591	0.9788	0.432	2.218	**0.003***	**1.8574**	**12.78**
**rs9268644**	A	C	**0.004265**	**1.886**	**1.218**	**2.921**	0.9672	1.012	0.5667	1.808	0.5969	1.118	0.7387	1.693	0.1256	1.489	0.894	2.481	**0.0322**	**1.3581**	**27.24**
**rs3129877**	A	G	**0.02308**	**1.706**	**1.074**	**2.711**	0.6702	1.151	0.6032	2.195	0.1675	1.375	0.8742	2.163	0.1798	1.479	0.834	2.625	**0.005***	**1.4506**	**0**
rs3135392	T	G	0.2571	1.282	0.834	1.971	0.7688	0.9146	0.5045	1.658	0.2361	1.291	0.8457	1.972	0.332	1.301	0.764	2.213	0.1047	1.2198	0
rs3177928	G	A	0.8042	1.102	0.5124	2.368	0.7893	1.158	0.3953	3.391	0.6259	1.172	0.619	2.219	**0.04049**	**0.382**	**0.148**	**0.9866**	0.7216	0.9146	29.09
**rs6937545**	A	C	**0.004038**	**1.887**	**1.222**	**2.914**	0.9734	1.01	0.5605	1.82	0.4446	1.182	0.7699	1.814	0.8744	0.9592	0.572	1.609	0.1674	1.2474	40.1

Abbreviations: NL, Non Lofgren sarcoidosis patient; LS, Sarcoidosis patient with Lofgren syndrome; NLR, Non Lofgren with resolved disease; NLP, Non Lofgren with persistent disease; C, controls

I, I2 heterogeneity index (0-100), low heteregeneity I<25, A1, allele 1; A2, allele 2

* Significant P values after multiple correction
