# Supplementary material for: SNP Variants in Major Histocompatibility Complex Are Associated with Sarcoidosis Susceptibility—A Joint Analysis in Four European Populations
Source: Front Immunol. 2017 Apr 19;8:422. doi: 10.3389/fimmu.2017.00422 (PMC5395694; doi:10.3389/fimmu.2017.00422)
Supplement: Supplementary file 3 [file Image_1.PDF]

Supplementary Material

SNP variants in MHC are associated with sarcoidosis susceptibility – a joint analysis in four European populations

Annika Wennerström[1†], Elisa Lahtela[1*†], Verneri Anttila[2,3,4], Martin Petrek[5], Johan Grunewald[6], Coline H.M. van Moorsel[7], Anders Eklund[6], Jan C Grutters[7], Vitezslav Kolek[5], Frantisek Mrazek[5], Amit Kishore[5], Leonid Padyukov[8], Anne Pietinalho[9], Marcus Ronninger[5], Mikko Seppänen[10], Olof Selroos[11], Marja-Liisa Lokki[1]

[1]Transplantation Laboratory, Medicum, University of Helsinki, Helsinki, Finland.

[2] Analytical and Translational Genetics Unit, Department of Medicine, Massachusetts General Hospital and Harvard Medical School, Boston, MA.

[3]Program in Medical and Population Genetics, Broad Institute of MIT and Harvard, Cambridge, MA, USA.

[4]Institute for Molecular Medicine Finland (FIMM), University of Helsinki, Helsinki, Finland.

[5] Faculty of Medicine and Dentistry and Institute of Molecular and Translational Medicine, Palacký University, Olomouc, the Czech Republic

[6]Respiratory Medicine Unit, Department of Medicine Solna and CMM, Karolinska Institutet and Karolinska University Hospital, Solna, Sweden

[7] Department of Pulmonology, St Antonius Hospital Nieuwegein, and Heart and Lung Center University Medical Center Utrecht, the Netherlands

[8]Rheumatology Unit, Department of Medicine, Karolinska Institutet and Karolinska University Hospital, Stockholm, Sweden

[9] Raasepori Health Care Centre, Raasepori, Finland;

[10] Immunodeficiency Unit, Division of Infectious Diseases, Department of Medicine, Helsinki University Central Hospital, Helsinki, Finland

[11] University of Helsinki, Helsinki, Finland

[†] A. Wennerström and L.E. Lahtela contributed equally to this paper.

*Correspondence:
Elisa Lahtela
Transplantation Laboratory, Medicum, University of Helsinki
P.O. Box 21
FI-00014 University of Helsinki
laura.lahtela@helsinki.fi
Telephone:+358-41-5482083

1 Supplementary Figures and Tables

1.1 Supplementary Figures

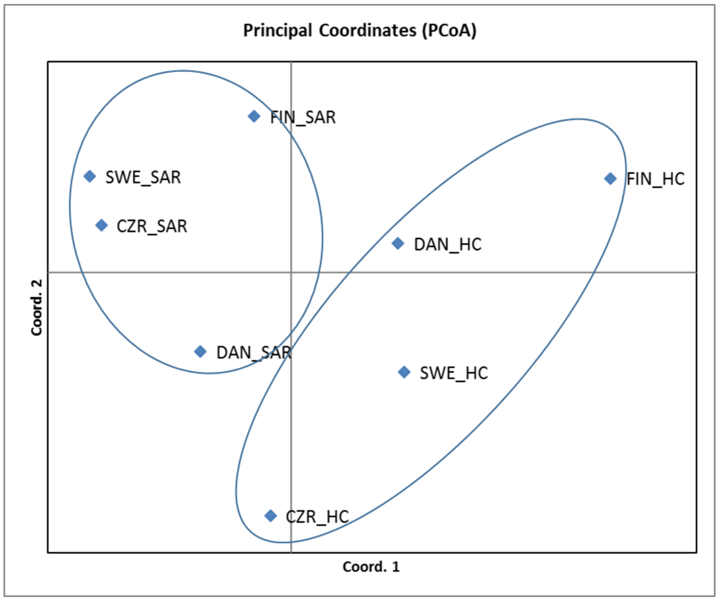

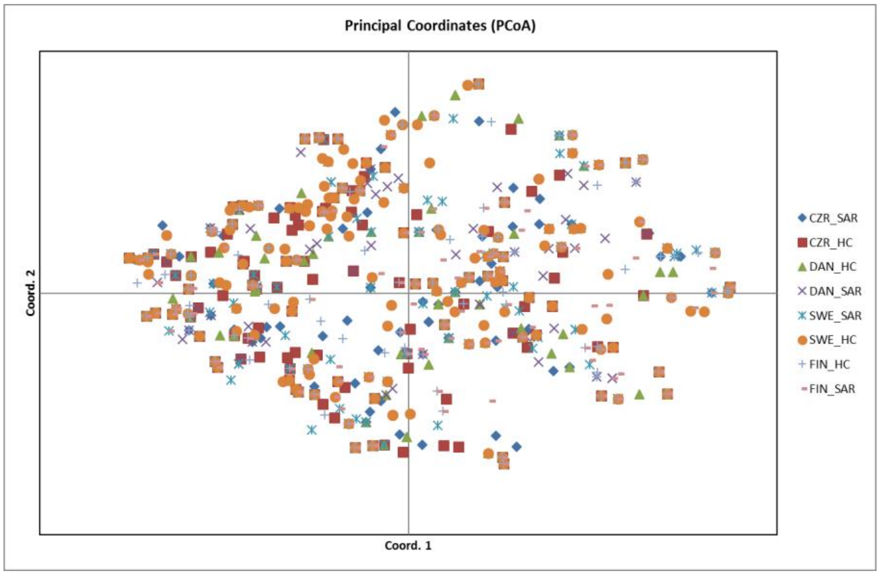

Supplementary Figure 1. Principal Coordinates analysis (PCoA) in Finnish, Swedish, Dutch and Czech sarcoidosis patients and controls. Figure above: PCoA using covariance matrix with data standardization from pairwise population Fst values from allele frequencies of analyzed loci.Figure below: PCoA using the linear genetic distance based covariance matrix with data standardization for the analyzed dataset.

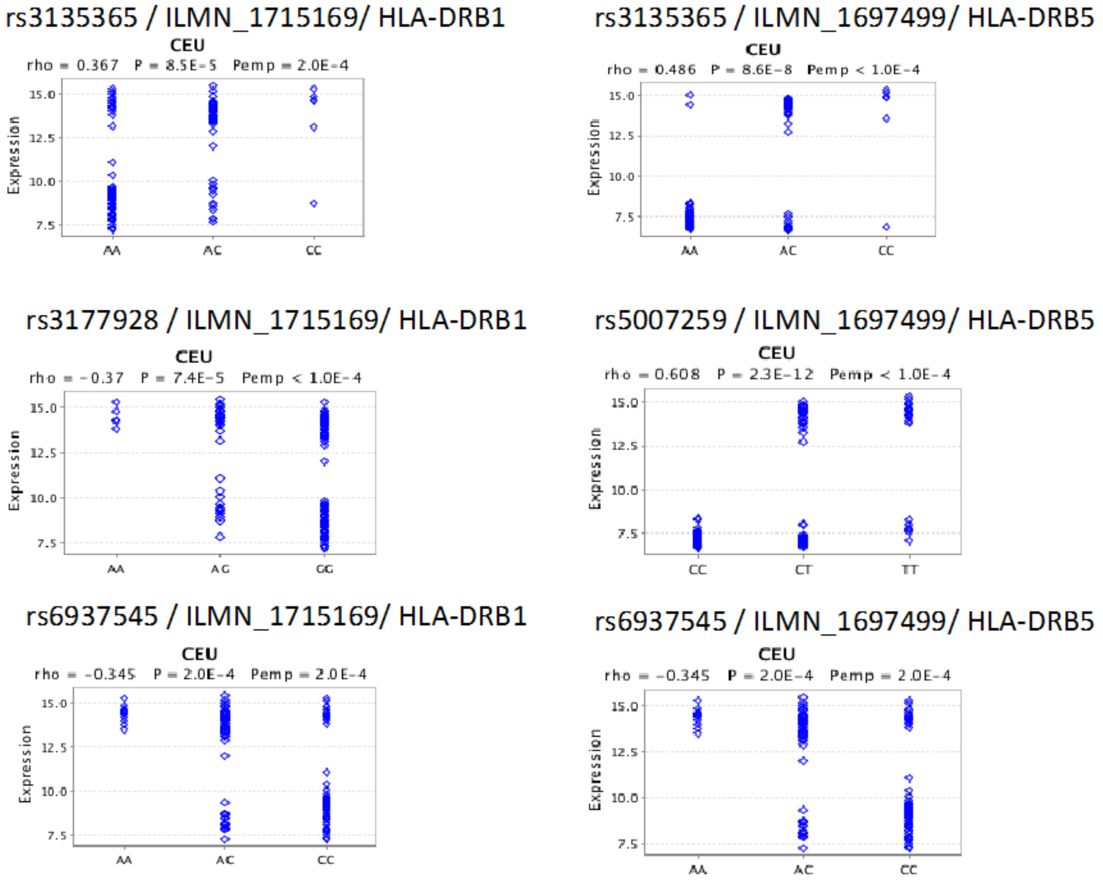

Supplementary Figure 2. SNP-probe association plots for SNPs rs3135365, rs3177928, rs6937545 and rs5007259. The GeneVar analysis presented that SNPs rs3135365, rs3177928, rs6937545 act as cis-acting eQTLs for HLA-DRB1, and rs3135365, rs6937545 rs5007259 as eQTLs for HLA-DRB5.
